# Deep Learning‐Powered Nanoplasmonic Biosensing Approach Enables Ultrasensitive Extracellular Vesicles Profiling for Cancer Screening

**DOI:** 10.1002/advs.202511337

**Published:** 2025-09-23

**Authors:** Jiaheng Zhu, Yingqi Xiao, Xinyue Huang, Qiang Niu, Lihuang Zeng, Shaowei Lin, Mengqi Jiang, Tianhao Huang, Hanyang Chen, Yinong Xie, Yuan Gao, Wei Chen, Yiming Yan, Jiaqing Shen, Kaibin Chen, Yurong Dai, Zhipeng Zhang, Lijun Zeng, Yahong Chen, Boan Li, Jinfeng Zhu, Bo Li

**Affiliations:** ^1^ Institute of Electromagnetics and Acoustics and Key Laboratory of Electromagnetic Wave Science and Detection Technology Xiamen University Xiamen 361005 China; ^2^ Department of Laboratory Medicine The Fifth Medical Center of Chinese PLA General Hospital Beijing 100039 China; ^3^ The First Affiliated Hospital of Xiamen University School of Medicine Xiamen University Xiamen 361003 China; ^4^ Department of Automation Tsinghua University Beijing 100084 China

**Keywords:** cancer screening, deep learning, extracellular vesicle, Kolmogorov‐Arnold network, metasurface, nanoplasmonic

## Abstract

Nanoplasmonic metasurface technology, known for its high sensitivity, has garnered significant attention in the field of cancer detection. However, its potential is currently hindered by the inefficient data processing and analysis of conventional biosensing approaches. Herein, a biosensing strategy based on the Kolmogorov–Arnold network (KAN)‐enabled metasurface chip (metaEVchip) for ultrasensitive small extracellular vesicles (sEV) analysis in serum is proposed. By analyzing full‐spectrum data from 600 pancreatic ductal adenocarcinoma (PDAC) patients and 1200 controls via KAN‐powered deep learning nanoplasmonic biosensing, the strategy achieves an exceptional area under the curve (AUC) of 0.99 in an external validation set, outperforming traditional methods. Further exploration of this enhanced performance reveals KAN's mechanism for the simultaneous capture of multi‐dimensional spectral features, an advantage that enables efficient data processing and accuracy. This advancement significantly expands the applicability of nanoplasmonic metasurfaces in biosensing and establishes a new paradigm for cancer screening and improved clinical management of multiple malignancies.

## Graphical abstract

This study presents a Kolmogorov–Arnold network (KAN)‐powered nanoplasmonic biosensing platform for ultrasensitive detection of small extracellular vesicles in serum, enabling highly accurate pancreatic cancer screening. The method achieves an area under the curve (AUC) of 0.99 in a large clinical cohort, offering a robust and interpretable deep learning framework for non‐invasive cancer diagnostics.

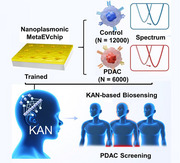

## 1 Introduction

Pancreatic ductal adenocarcinoma (PDAC), one of the deadliest cancers, typically presents as advanced and unresectable disease, with a 5‐year survival rate of only 12%. Early detection could improve survival rates by 30%–40%.^[^ ^1^, ^2^, ^3^, ^4^, ^5^ ^]^ Currently, the detection of carbohydrate antigen 19‐9 (CA 19‐9) levels in serum is the most widely used liquid biopsy for PDAC, but it lacks sensitivity and specificity, leading to potential misdiagnosis or missed diagnosis.^[^ ^6^, ^7^ ^]^ These limitations highlight the need for more reliable diagnostic markers. As a promising alternative, detecting small extracellular vesicles (sEV) in serum offers an excellent non‐invasive diagnostic approach.^[^ ^8^, ^9^, ^10^ ^]^ sEV are nanoscale membrane vesicles actively secreted by a wide range of mammalian cells, particularly rapidly dividing cancer cells.^[^ ^11^, ^12^, ^13^ ^]^ These vesicles are abundant in the blood and are crucial in mediating intercellular communication. The surface of tumor‐derived sEV is enriched with specific proteins compared to healthy controls (HC).^[^ ^14^, ^15^, ^16^ ^]^ For example, GPC1‐ and EphA2‐positive sEV exhibit high specificity and sensitivity in distinguishing PDAC from healthy controls and patients with other diseases.^[^ ^17^, ^18^, ^19^, ^20^ ^]^ These tumor‐specific markers can potentially overcome the limitations of CA 19‐9, offering a more sensitive and specific diagnostic tool for PDAC.

In this context, nanoplasmonic metasurfaces, leveraging the strong localized electric field enhancement at resonant wavelengths, have emerged as a powerful tool for label‐free and non‐destructive identification of various biological samples.^[^ ^21^, ^22^, ^23^, ^24^ ^]^ Specifically, the intrinsic properties of analytes induce alterations in the dielectric environment, which are manifested as wavelength shifts and intensity changes in the metasurface response. These changes are highly correlated with the concentration of the analytes, thereby offering the opportunity for enabling precise and sensitive detection.^[^ ^25^, ^26^, ^27^ ^]^ In addition, the refractive effect (RE), the spectral effect (SE), and the nanoplasmonic loading effect (NLE) each analyze the spectral response from different perspectives to elucidate the basis for determining analyte concentration and type.^[^ ^28^, ^29^ ^]^ However, reliance on a single sensing perspective alone can lead to the loss of valuable information, especially for nanoplasmonic spectra with complex and large data, thereby diminishing the effectiveness of nanoplasmonic biosensing.

In contrast, Artificial Intelligence (AI) can extract data features from multiple domains, enabling enhanced pattern recognition from nanoplasmonic spectra and improving the efficiency of nanoplasmonic biosensing.^[^ ^30^, ^31^, ^32^, ^33^, ^34^, ^35^ ^]^ For sEV spectral analysis, effectively capturing subtle, continuous spectral shifts and interpreting the contributions of multi‐dimensional features remains challenging due to the high‐dimensional, continuous, and physically correlated nature of nanoplasmonic responses. To address this, we employed the Kolmogorov–Arnold network (KAN), which adaptively learns optimized activation functions through spline‐based transformations. This approach offers superior capability in modeling spectral continua and explicitly deciphering how individual wavelength regions contribute to prediction—overcoming key limitations of conventional CNNs or Transformers, which often suffer from inflexible inductive biases or lack inherent interpretability when handling such complex spectral data (see more details in Table S1, Supporting Information).

**Figure 1:**
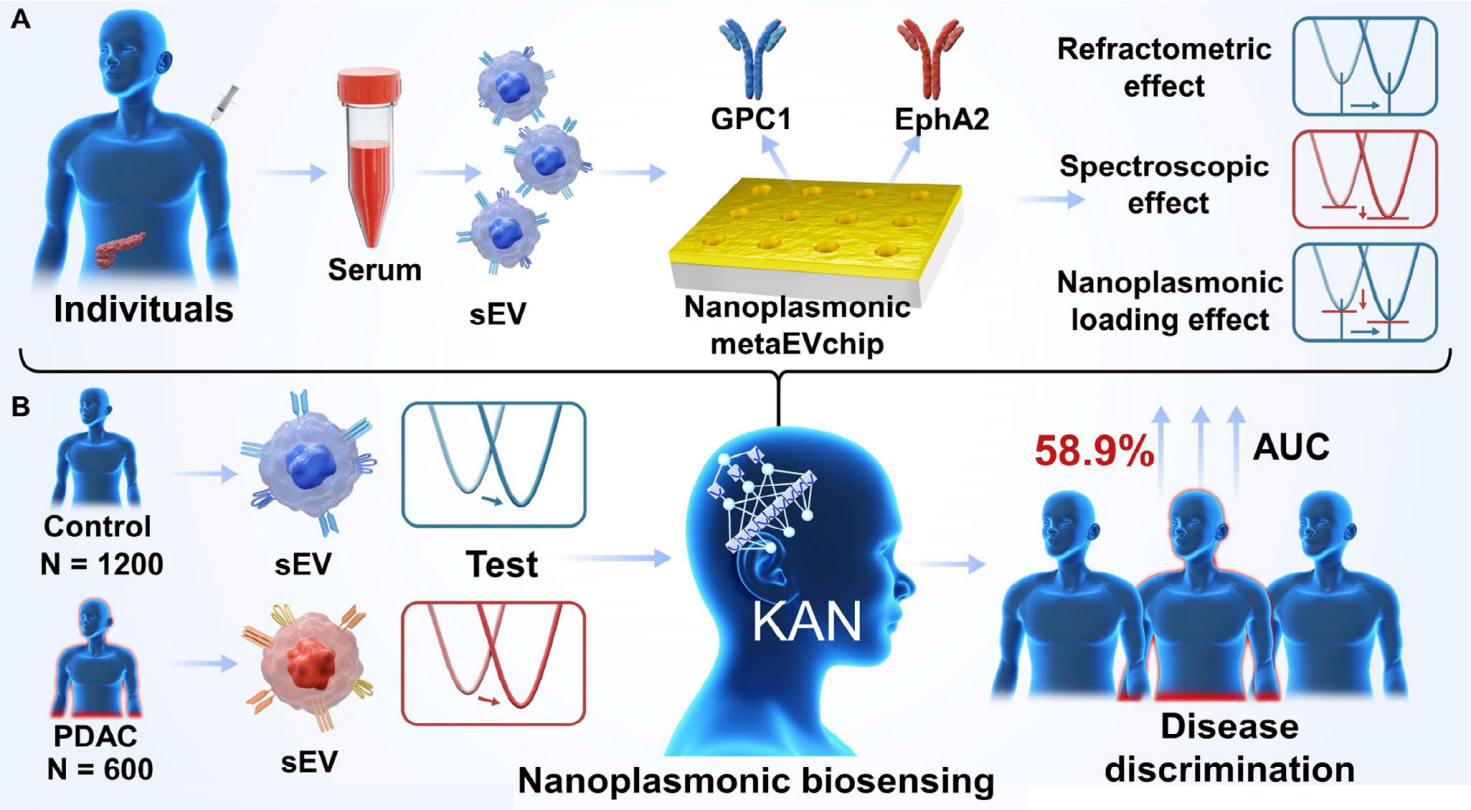
Schematic drawing of KAN‐powered nanoplasmonic biosensing approach for sEV detection to facilitate PDAC screening. A) Two membrane proteins, GPC1 and EphA2, specific to PDAC‐derived sEV, are detected using the nanoplasmonic metaEVchip. This results in a large number of nanoplasmonic spectra, including contributions from the RE, SE, and NLE. B) KAN deep learning, trained by nanoplasmonic spectroscopy, enhances the diagnostic performance of PDAC screening.

Here, we propose a nanoplasmonic biosensing method based on the KAN deep learning for high‐sensitivity detection of sEV in serum to facilitate PDAC screening. We develop a wafer‐scale periodic nanoplasmonic gold nanohole chip for targeted high‐throughput detection of two PDAC‐specific sEV membrane proteins (GPC1 and EphA2) in clinical serum, as shown in **Figure** **1**A. The acquired spectral information is further subjected to global analysis using KAN deep learning, as illustrated in Figure 1B. In this process, the model analyzes the entire spectrum to classify each signal as either a control or a PDAC, specifically highlighting the evolution of three key effects. In the experiment, the study cohort comprised 600 PDAC patients and 1200 controls, including an independent external validation set (120 PDAC/240 controls) for assessing the diagnostic performance of this sensing modality in PDAC. The control group includes 900 HC, 100 with pancreatitis, 100 with cholangiocarcinoma (CCA), and 100 with hepatocellular carcinoma (HCC). The model outputs are compared with the results of individual and combined analyses of all resonance wavelength positions under the conventional nanoplasmonic sensing approach, reflecting better diagnostic results. Notably, the KAN‐powered nanoplasmonic biosensing approach achieves a 58.9% higher area under the curve (AUC) compared to conventional methods. These results highlight the enhanced sensing performance of the KAN‐driven deep‐learning nanoplasmonic sEV platform for PDAC detection. Furthermore, the methodology developed in this study could be extended to tumor‐specific sEV analysis in other cancer screening applications.

## 2 Results and Discussion

### 2.1 Wafer‐Scale fabrication and Nanoplasmonic Effects of metaEVchip

**Figure 2:**
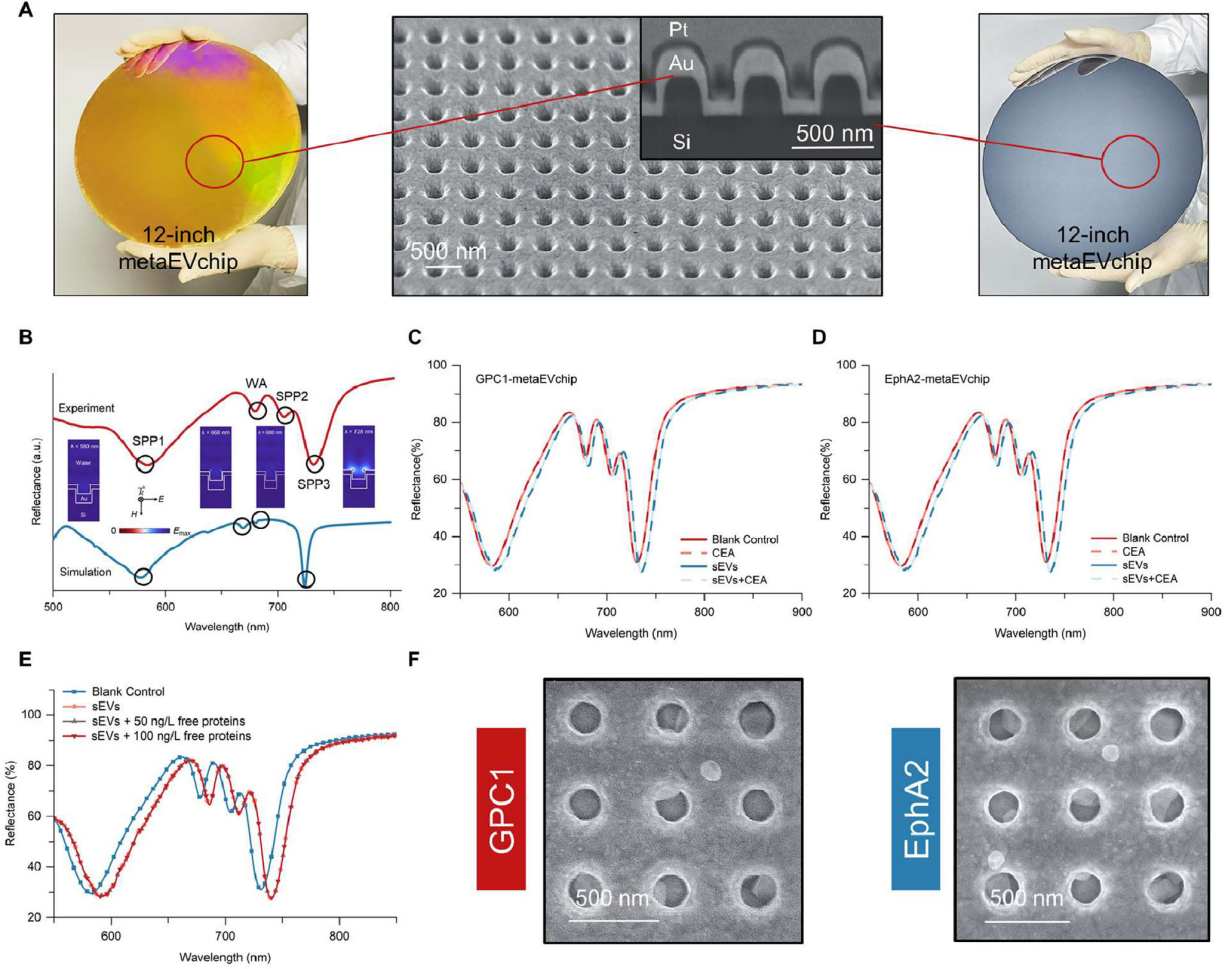
Nanoplasmonic metaEVchip's wafer‐scale fabrication and performance testing. A) Wafer‐scale nanoplasmonic gold nanoholes are fabricated on a 12‐inch Si wafer. The fabricated wafer is diced into 3 mm2 chips for biomarker assays. SEM of the metaEVchip sensor chip shows the periodic quadrilateral lattice of gold nanohole structures. B) Experimental and simulated reflectance spectra of metaEVchip, where inserts show the electric field distribution corresponding to the four optical modes. Binding specificity tests for C) the GPC1‐metaEVchip and D) the EphA2‐metaEVchip. E) Detection results of adding different concentrations of GPC1 free proteins to sEV on the reflectance spectra using GPC1‐metaEVchip. F) SEM images showing metaEVchip specifically capturing sEV from serum via functionalized antibody GPC1 and antibody EphA2.

The nanoplasmonic metasurface chip (metaEVchip) is engineered for clinical‐scale biosensing applications, leveraging its high‐throughput detection capability to enable efficient processing of large sample sizes. This biosensor features periodic gold nanopore arrays fabricated on twelve‐inch wafers using nanoimprint lithography (see Experimental Section). Scanning electron microscopy (SEM) characterization confirms the array parameters with 200 nm diameter nanopores arranged in 500 nm periodic spacing and 200 nm depth (**Figure** **2**A). The designed nanostructure geometry enables dual optical phenomena upon illumination: surface plasmon polariton (SPP) excitation and Wood‐Rayleigh anomaly (WA) generation. The resonance wavelength of the SPP can be quantitatively analyzed using Equation (1):^[^ ^36^, ^37^ ^]^

(1) $$\lambda_{spp}=\frac{P}{\sqrt{\left(i^{2}+j^{2}\right)}}\;\sqrt{\frac{\varepsilon_{d}\varepsilon_{Au}}{\varepsilon_{d}+\varepsilon_{Au}}}\;$$

where *P* represents the center‐to‐center spacing of adjacent nanoholes; *i* and *j* are integers denoting the order numbers of the reciprocal quadrilateral lattice vector; *ε_Au_* is the permittivity of gold; and *ε_d_* is the refractive index of the environmental dielectric material. While the WA can be evaluated using Equation (2):^[^ ^38^ ^]^

(2) $$\lambda_{WA}=\frac{P}{\sqrt{\left(i^{2}+j^{2}\right)}}\;\sqrt{\varepsilon_{d}}\;$$

Figure 2B shows that four optical modes are generated in both experiments and simulations: a mode at 665 nm corresponding to the WA, which is primarily determined by the metaEVchip periodicity and is insensitive to structural variations; a mode at 680 nm representing an SPP at the gold‐silicon interface, with field strength concentrated at this interface; and modes at 580 and 728 nm corresponding to SPP at the gold‐water interface. Based on field strength simulations and previous studies,^[^ ^39^ ^]^ the SPP3 mode exhibits concentrated field strength at the gold‐water interface, facilitating sensitive detection of analytes on the metal surface.

To systematically evaluate the detection performance of the metaEVchip biosensor, Figure 2C demonstrates that only a weak response is obtained when the GPC1‐metaEVchip is exposed to a non‐target protein (CEA: carcinoembryonic antigen), while significant spectral changes are detected upon interaction with sEV (purified from PDAC patients via ultracentrifugation). When detecting analytes containing sEV and CEA in a mixture, the metaEVchip shows exceptional nanoplasmonic spectral stability even in the high concentrations of interfering scrambled proteins (>1 mg mL^−1^). As shown in Figure 2D, the EphA2‐metaEVchip exhibits a similar phenomenon. Even when different concentrations of free proteins are added during sEV detection, the SPP3 mode, which has the best sensing effect, shows no spectral response. This indicates that the metaEVchip is specifically responsive to sEV (Figure 2E). In clinical serum assays (Figure 2F), both the GPC1‐metaEVchip and EphA2‐metaEVchip successfully capture sEV from serum, with specific binding near the gold nanoholes. These results demonstrate the nanoplasmonic metaEVchip's promise and clinical sufficiency for sensing GPC1 and EphA2 sEV.

### 2.2 Nanoplasmonic metaEVchip Conventional Biosensing Mechanisms for sEV

**Figure 3:**
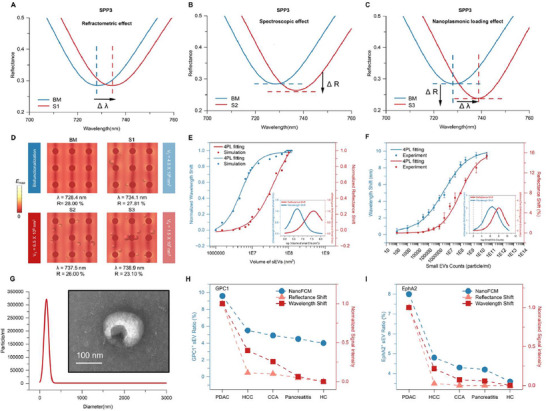
Simulation and experimental validation of nanoplasmonic metaEVchip to detect different numbers of sEV. Nanoplasmonic metaEVchip detects sEV based on conventional biosensing mechanisms: A) RE: wavelength shift due to the refractive index of the sEV; B) SE: reflectance shift due to absorption of the sEV; and C) NLE: The modulation of electromagnetic fields and resonance modes resulting from the binding of sEV to the surface of metaEVchip, i.e., changes in wavelength and reflectance intensity. D) Distribution of field strength corresponding to the detection of different numbers of sEV by metaEVchip. The wavelength shift and reflectance shift corresponding to metaEVchip detection of different numbers of sEV are represented by both E) simulation and F) experiment, with insets showing the differentiation for the resonance shift and the reflectance shift. Initially, the resonance shift is more sensitive to changes in the number of sEV, producing a rapid response. As the number of sEV increases, the reflectance shift becomes dominant in responding to changes in sEV concentration. This transition reflects the dynamic interplay between the RE, SE, and NLE during sEV detection. G) Particle size distribution of sEV by NTA, where the inset shows their representative morphology by TEM. SEV detection of PDAC, HCC, CCA, pancreatitis, and HC samples based on the NanoFCM, H) GPC1 metaEVchip, and I) EphA2 metaEVchip, respectively.

Upon entering the highly confined electromagnetic field near the metaEVchip on a subwavelength scale, sEV engage in a complex interplay characterized by three distinct yet simultaneous effects: RE, SE, and NLE.^[^ ^40^ ^]^ To explore the responses of these three effects during sEV detection, we employ optical simulations to analyze the low‐order nanoplasmonic mode SPP3. **Figure** **3**A–C illustrates the spectral responses of RE, SE, and NLE induced by different numbers of sEV on the nanoplasmonic metaEVchip. Specifically, as shown in Figure 3D (BM, S1), when the number of sEV is low, the dominant effect is RE. According to Equation (3), the wavelength shift is induced by the change in the refractive index Δn of the surface:^[^ ^41^ ^]^

(3) Δλ=S×Δn

where *S* stands for metaEVchip's sensitivity to refractive index. Considering Langmuir adsorption kinetics, the surface coverage of sEV θ is given by:^[^ ^42^ ^]^

(4) $$\theta =\frac{Kx}{1+Kx}$$

where *K* is the Langmuir equilibrium constant and *x* is the sEV concentration. The equivalent refractive index upon sEV binding is calculated as:^[^ ^43^ ^]^

(5) $$n=\theta \times n_{sEV}+\left(1-\theta \right)\times n_{water}\;$$

At low sEV concentrations, binding leads to a significant change in the local refractive index, resulting in a pronounced resonance wavelength shift.

As the sEV concentration increases (Figure 3D (S3,S4)), the surface gradually saturates, and the rate of refractive index change slows down. While the effect of localized refractive index variations on the resonance wavelength modulation is reduced, the cumulative adsorption of sEV particles emerges as the dominant light‐scattering mechanism, leading to changes in light intensity. According to the Beer‐Lambert law:^[^ ^44^ ^]^

(6) A=εcl

where ε is molar absorptivity, absorbance (*A*) is proportional to the concentration of molecules *c* and the optical path length *l*. In our sensing system, the optical path length is fixed and determined by the near‐field limit of the nanoplasmonic metasurface (ranging from hundreds to thousands of nanometers). Thus, the concentration of sEV is reflected in the intensity of the reflectance spectrum. As the concentration increases, absorbance rises, leading to a significant decrease in reflected light intensity. At this point, the wavelength shift diminishes in significance, while SE, dominated by intensity changes, starts to dominate. Meanwhile, NLE accounts for both changes. It refers to the phenomenon where the response of a metasurface during biomolecule detection is similar to that of a nanoplasmonic antenna during load changes, resulting in simultaneous changes in resonance wavelength and intensity. As shown in Figure 3E,F, both simulations and experiments confirm our theoretical derivation: when the number of sEV is low, the wavelength shift dominates, responding quickly to changes in sEV number. Conversely, when the number of sEV is high, the reflectance intensity change exceeds the wavelength shift, dominating the response. Therefore, in practical sEV nanoplasmonic biosensing applications, differential responses to wavelength shifts and reflection intensity variations emerge across sEV concentration gradients. This phenomenon is characterized by dynamic reconfigurations of RE, SE, and NLE. As shown in Figure 3G, we ultracentrifuge clinical serum samples to isolate sEVs (sizes< 200 nm) and detect GPC1 and EphA2 using nanoflow cytometry (NanoFCM). Figure 3H,I demonstrates that the nanoFCM results are in good agreement with both the reflectance shift and the wavelength shift. These results confirm that the metaEVchip can effectively measure sEVs, thereby ensuring its reliability and potential for clinical application.

### 2.3 Conventional Nanoplasmonic Biosensing in Large Clinical Sample Amounts

**Figure 4:**
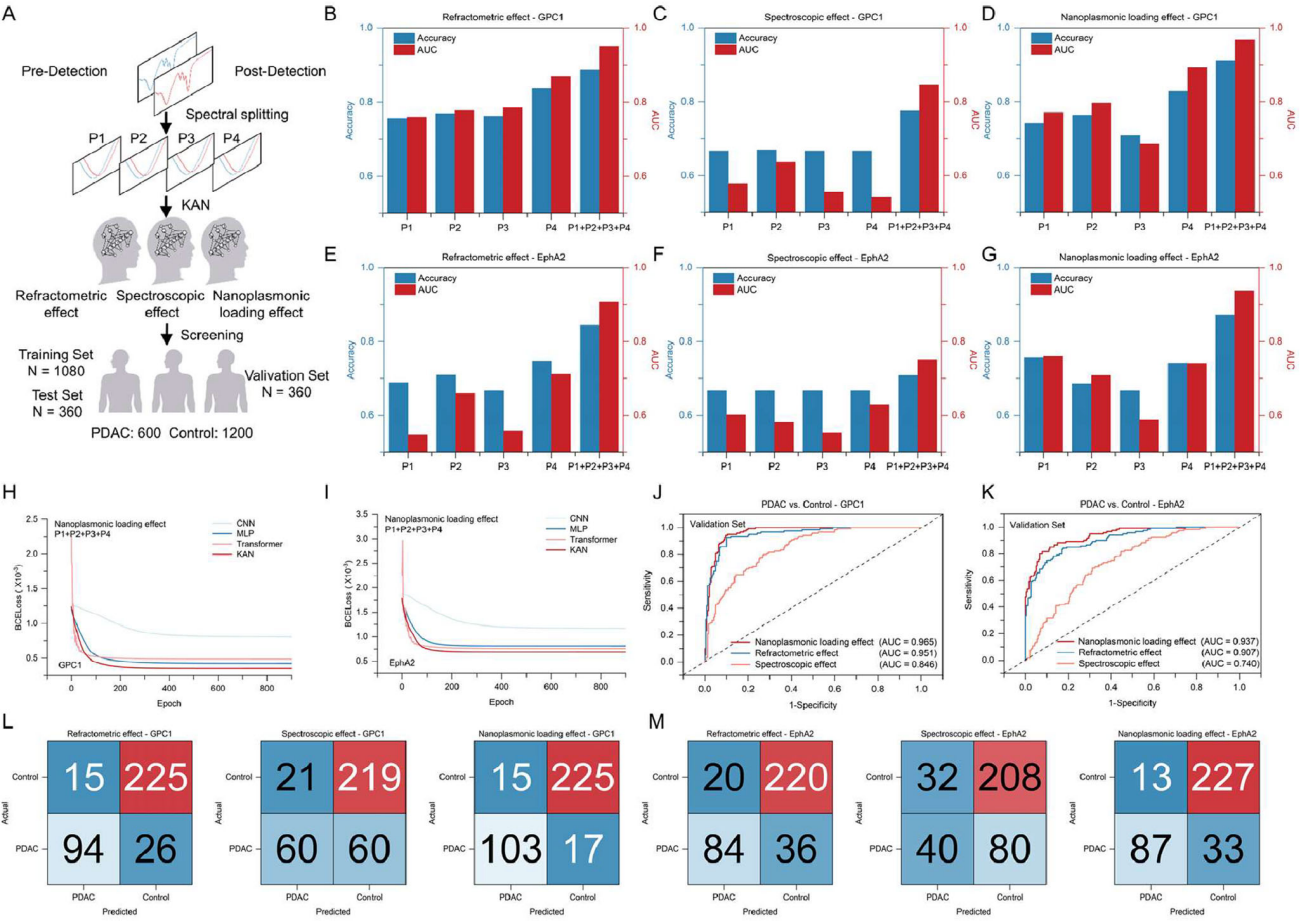
Performance analysis of conventional nanoplasmonic biosensing in clinical sample testing. A) Schematic analysis of the changes of the four modes in the pre‐ and post‐detection nanoplasmonic spectra by three conventional nanoplasmonic biosensing methods (RE, SE, NLE), combined with KAN deep learning to achieve the screening of PDAC. Accuracy and AUC performance for B) RE, C) SE, and D) NLE under GPC1‐metaEVchip per mode and four‐mode fusion in the KAN deep learning model. Accuracy and AUC performance for E) RE, F) SE, and G) NLE under EphA2‐metaEVchip per mode and four‐mode fusion in the KAN deep learning model. Learning curves of convolutional neural network (CNN), multilayer perceptron (MLP), Transformer, and KAN in NLE biosensing with H) GPC1‐metaEVchip and I) EphA2‐metaEVchip. receiver operating characteristic (ROC) curves of four mode fusions in J) GPC1 and K) EphA2 for RE, SE, and NLE. Four mode fusions in the confusion matrix of L) GPC1 and M) EphA2 for RE, SE, NLE.

Here, we collected nanoplasmonic spectra of GPC1‐metaEVchip and EphA2‐metaEVchip, both before and after detecting clinical serum samples. Serum samples from 600 PDAC patients and 1200 controls have their diagnoses confirmed by pathology and are stored according to the routine protocol at each medical center. In the conventional sensing approach for detecting clinical samples using nanoplasmonic metaEVchip, the sensing analysis of analytes is primarily performed at the nanoplasmonic mode with the best sensing effect.^[^ ^45^ ^]^ In contrast, recent studies have shown that combining multi‐dimensional sensing signals using machine learning algorithms can improve sensing performance.^[^ ^46^, ^47^, ^48^ ^]^ As shown in **Figure** **4**A, we spectrally split the acquired nanoplasmonic spectra into four modes (P1, P2, P3, P4) and combined them with three biosensing modes (RE, SE, NLE) in the KAN deep learning model to screen for PDAC.

As depicted in Figure 4B, for RE, P4 exhibits the best sensing effect, outperforms the other three modes (P1, P2, P3) in terms of detection accuracy and diagnostic performance AUC in clinical samples. Combining all four modes further improves PDAC screening performance. Similarly, in SE and NLE (Figure 4C,D), the best performance is achieved by integrating the four modes. This trend is consistent when using the EphA2‐metaEVchip (Figure 4E–G). Therefore, we evaluate the KAN neural network performance in NLE mode by combining the four modes. The loss function is defined by calculating the difference between the model output and the real labels (BCEloss) as follows:^[^ ^49^ ^]^

(7) $$BCEloss\;\left(y,\hat{y}\right)=-\frac{1}{N}\sum_{i=1}^{N}\left(y_{i}\log\left({\hat{y}}_{i}\right)+\left(1-y_{i}\right)\log\left(1-{\hat{y}}_{i}\right)\right)\;$$

where *N* is the number of samples, *y_i_* is the real label of the ith sample (0 or 1), and ${\hat{y}}_{i}$ is the predicted value of the ith sample. As shown in Figure 4H,I, compared with other deep learning algorithms, KAN achieves the lowest BCEloss in GPC1 and EphA2 by using the lowest training parameters in detecting sEV in clinical sample serum (see Figures S2–S4, Supporting Information). Similarly, in both RE and SE, KAN achieves the lowest BCEloss with the lowest training parameters and number of iterations, implying that the KAN neural network has better diagnostic performance than other traditional deep learning models. Further, in the independent validation cohort (*n* = 360), Figure 4J,K shows that NLE achieves the best AUC in both GPC1 and EphA2 detection, and we can also intuitively find that NLE has the best PDAC screening accuracy through the confusion matrix in Figure 4L,M. Considering that RE and SE focus on wavelength shift or reflectance shift alone, NLE focuses on both wavelength shift and reflectance shift, thus achieving better sensing performance in high‐throughput nanoplasmonic biosensing. This confirms that combining multiple sensing information is a promising approach to overcome the limitations of relying on a single biosensing effect in cancer screening.

### 2.4 KAN‐Powered Nanoplasmonic Biosensing in Large Clinical Sample Amounts

**Figure 5:**
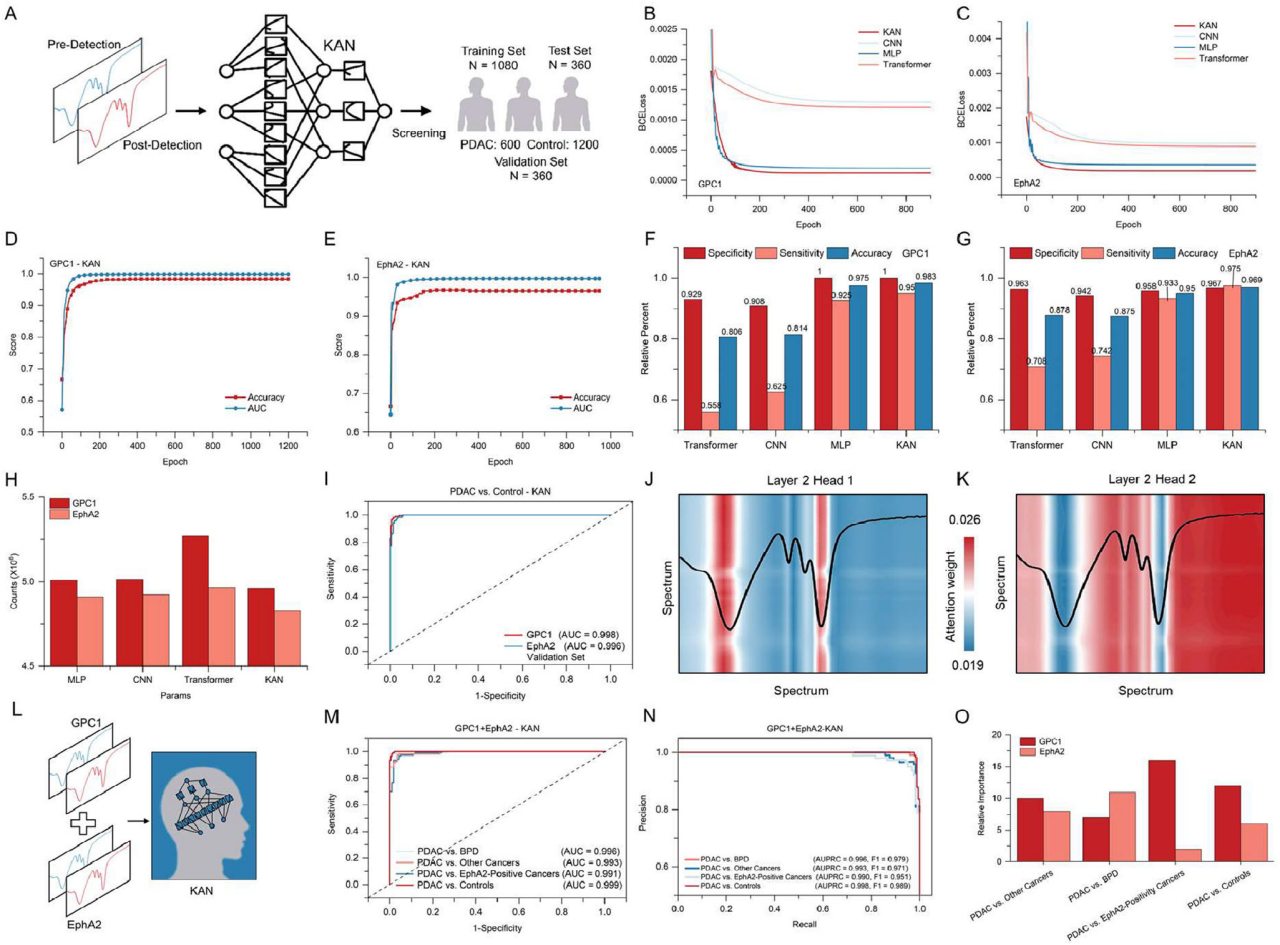
Diagnostic performance analysis of KAN‐powered nanoplasmonic biosensing in PDAC screening. A) Schematic of the screening of PDAC by feeding the nanoplasmonic spectra into the KAN deep learning. Learning curves of CNN, MLP, Transformer, and KAN in deep learning‐powered biosensing with B) GPC1‐metaEVchip and C) EphA2‐metaEVchip. AUC and accuracy curve of the training process with D) GPC1‐KAN and E) EphA2‐KAN. Specific sensitivity and accuracy of different deep learning models in the detection of F) GPC1 and G) EphA2. H) The number of parameters required for the training process of different deep learning models. I) KAN‐powered biosensing detects the ROC curves corresponding to GPC1 and EphA2. J,K) Heatmap of attentional weights for various network layers and multiple heads during KAN‐powered biosensing. L) Diagram of combined GPC1 and EphA2 for PDAC screening via KAN deep learning. M) ROC curve of GPC1 combined with EphA2 under KAN‐powered biosensing. N) PRC curve for the entire test set. O) Relative importance corresponding to GPC1 and EphA2 in the diagnostic process during the combined GPC1 and EphA2 assay.

In order to further enhance the performance of nanoplasmonic biosensing, as shown in **Figure** **5**A, we feed the entire nanoplasmonic spectra before and after detecting clinical serum samples into the KAN for learning. This approach ensures that no potential sensing information in the nanoplasmonic spectra is overlooked. Unlike conventional approaches that segment spectra based on localized resonance features (Figure S8, Supporting Information), our method retains full‐spectrum characteristics for enhanced pattern recognition. Figure 5B shows that KAN converges to the lowest BCEloss of ≈1.33 × 10^−^⁴ in the test instance when detecting GPC1‐sEV in clinical serum samples, requiring fewer epochs than the other three models (see Figure S7, Supporting Information). Meanwhile, Figure 5C demonstrates that the KAN deep learning model also exhibits the best sensing performance in high‐throughput detection of EphA2. This is attributed to the KAN model's design based on the Kolmogorov–Arnold representation theorem, which decomposes complex multivariate functions into combinations of univariate functions. This design enables KAN to better adapt to the complex data distribution in tasks requiring high‐precision fitting.

As shown in Figure 5D,E, with increasing epoch, the AUC and accuracy quickly reach stable values in the detection of GPC1 and EphA2. This indicates that the KAN deep learning model rapidly extracts a large amount of useful information from the nanoplasmonic spectra in the validation set. To further elucidate the screening performance of KAN‐powered nanoplasmonic biosensing for detecting sEV in serum against PDAC, Figure 5F,G shows that the KAN deep learning model achieves the best specificity, sensitivity, and accuracy compared to other deep learning models. Notably, a screening accuracy of up to 98.3% is achieved for EphA2 detection. Additionally, Figure 5H shows that KAN requires fewer parameters than the other three models in this process. As shown in Figure 5I, we further evaluate the diagnostic performance of the KAN‐powered deep learning model using ROC curves, with AUCs reaching 0.998 and 0.996 for the detection of the sEV membrane proteins GPC1 and EphA2, respectively. To explore why the KAN‐powered deep learning model achieves high diagnostic performance in nanoplasmonic biosensing and to elucidate its learning mechanism, we introduce the multi‐head attention mechanism and position encoding (see Figure S10, Supporting Information for details). These techniques ensure that the model acquires positional information of the equidistant exciton spectral lines, calculated as follows:^[^ ^50^ ^]^

(8) $$\frac{pos}{10000^{\frac{2i}{d_{model}}}}=e^{\left(\log\left(pos\right)-\frac{2i}{d_{model}}\log\left(10\;000\right)\right)}$$

where *pos* is the position of the spectral patch in the sequence, *d_model_* denotes the embedding dimension, and *i* is the dimension index.

Figure 5J,K displays the learning heatmaps of KAN‐powered nanoplasmonic biosensing, which illustrate multiple attention weights across representative layers and heads. In Figure 5J, head 1 in layer 2 highlights four regions with high attentional weights, all located at the positions of the nanoplasmonic resonance wavelength. This indicates that KAN deep learning in nanoplasmonic biosensing focuses on these four nanoplasmonic modes and assigns higher attentional weights to the significant resonance wavelength positions of both higher‐order and lower‐order nanoplasmonic modes. These highlighted regions demonstrate self‐attention to each mode and mutual attention among the four modes. This aligns with our conventional nanoplasmonic biosensing approach, which emphasizes spectral line changes at the resonance wavelength positions. In Figure 5K, head 2 of layer 2 shows five highlighted regions focusing on spectral line positions beyond the resonance wavelength troughs. Notably, attention is also given to changes near the half‐width positions of both higher‐order and lower‐order nanoplasmonic modes. These results demonstrate that the KAN neural network not only focuses on changes at the resonance wavelength positions but also on the overall changes in the nanoplasmonic spectral lines. This elucidates the attention to spectral details in the KAN‐powered nanoplasmonic biosensing approach and explains why KAN‐powered nanoplasmonic biosensing outperforms conventional methods in PDAC screening (see **Table** **1**).

**Table 1:** Performance comparison between KAN‐powered biosensing and other conventional biosensing on PDAC versus Controls.

Nanoplasmonic Biosensing	Accuracy [GPC1/EphA2]	AUC [GPC1/EphA2]
Refractometric effect	0.886/0.844	0.950/0.907
Spectroscopic effect	0.775/0.707	0.846/0.749
Nanoplasmonic loading effect	0.911/0.872	0.966/0.938
KAN‐powered deep learning	0.983/0.969	0.998/0.996

For PDAC detection, both GPC1 and EphA2 exhibit high accuracy and AUC values. Recent studies have shown that combining clinical information can enhance diagnostic performance.^[^ ^51^, ^52^ ^]^ To validate the specificity of the combined GPC1 and EphA2 assay in distinguishing PDAC from other EphA2‐positive cancers, we introduce an additional cohort of 100 cases comprising other EphA2‐positive malignancies (30 breast cancer and 70 colorectal cancer samples). As summarized in Table S3 (Supporting Information), GPC1 serves as a specificity anchor owing to its near‐exclusive expression in PDAC, which effectively minimizes false positives that could arise from other EphA2‐positive cancers, such as breast cancer. Concurrently, EphA2 acts as a sensitivity amplifier by capturing early‐stage PDAC cases that may show heterogeneous GPC1 expression. This dual‐marker strategy ensures broad diagnostic coverage across patient populations while maintaining high accuracy (Figure 5L). Figure 5M demonstrates that the combined use of GPC1 and EphA2 not only effectively distinguishes PDAC from controls but also maintains high specificity when differentiating PDAC from other EphA2‐positive cancers, achieving an AUC of 0.991. Furthermore, this dual‐marker approach exhibits outstanding diagnostic performance in discriminating PDAC from benign pancreatic diseases (BPD) and from other cancer types. These results are corroborated by the precision–recall curves (PR‐AUC) in Figure 5N, which confirm the strong predictive capacity of the GPC1+EphA2 combination for positive PDAC cases.

The two markers operate not in a simple additive manner but form a dynamic specificity‐sensitivity matrix. The KAN continuously re‐optimizes its relative contributions in response to spectral inputs (Figure 5O). GPC1 establishes a precise diagnostic direction, while EphA2 extends the detection range, resulting in a context‐aware classification system that remains highly adaptive and reliable across diverse clinical samples.

## 3 Conclusion 

In this study, we introduce a deep learning‐enhanced nanoplasmonic biosensing platform based on KAN for the detection and analysis of sEVs in large clinical serum samples. Although nanohole‐based surface plasmon resonance sensing is an established technology, conventional approaches remain limited by single‐parameter detection and poor manufacturability, hindering clinical adoption. Our metaEVchip overcomes these constraints through wafer‐scale fabrication, enabling high‐throughput clinical screening, and integrates deep learning to capture complementary nanoplasmonic features—significantly improving diagnostic accuracy (see more details in Table S2, Supporting Information). The metaEVchip exhibits distinct spectral shifts in response to varying sEV concentrations, a behavior validated through theoretical modeling and simulations. We further demonstrate experimentally that incorporating multi‐dimensional sensing information substantially enhances both the sensitivity and diagnostic performance of sEV detection. By leveraging KAN‐powered deep learning to analyze full nanoplasmonic spectra, we ensure comprehensive feature utilization and provide interpretable insights into the learning process. In a large‐scale clinical validation, the platform successfully distinguished patients with PDAC from controls with high specificity and sensitivity, outperforming conventional diagnostic methods. These results underscore the potential of our approach to advance nanoplasmonic biosensing technology and establish a new paradigm for accurate, efficient, and scalable cancer screening. Although the current study focuses on GPC1 and EphA2, the modular and adaptive design of our KAN‐driven framework allows seamless integration of new biomarkers like SLC5A3 to further improve diagnostic specificity.^[^ ^53^, ^54^, ^55^, ^56^ ^]^

## 4 Experimental Section

### Fabrication of Nanoplasmonic metaEVchip

The fabrication of metaEVchip involved the following key steps: First, a flexible polymer substrate (Obducat AB, Lund, Sweden) was used to replicate nanopore arrays from a nickel mold via thermal nanoimprint lithography. The imprinting process was conducted at 150 °C and 40 bar pressure. Next, silicon wafers coated with TU‐170 UV resist (Lund, Sweden) were imprinted with polymer nanopatterns at 65 °C and 30 bar pressure for 1 min to transfer the patterns onto the resist layer. Subsequently, the wafers were etched using a deep etching system (AMS200, Alcatel, France) in a sulfur hexafluoride and C_4_F_8_ (3:2) atmosphere for 35 s. Finally, a 3 nm chromium layer followed by a 150 nm gold layer was deposited on the wafers via sputtering (DISC‐SP‐3200, Genesis Technology, Beijing, China). The wafers were then diced into 3 × 3 mm^2^ pieces for nanoplasmonic metaEVchip preparation.

### Optical Measurement of metaEVchip

Optical measurements of the metaEVchip were conducted using a spectrometer with an integrated fiber optic probe (Avantes BV, Apeldoorn, The Netherlands). The probe comprised six illumination fibers and one reading fiber, the latter featuring a 200 µm core diameter. During reflectance spectroscopy measurements, the probe was positioned vertically 4 mm above the MetaEVchip surface. A gold mirror served as the spectral reference for calibration. For sEV assays, the incubation microchamber was primed with deionized water (DW).

### Functionalization of Antibody on metaEVchip

The metaEVchip is functionalized via sulfate modification and antibody immobilization. The antibodies used were anti‐GPC1 and anti‐EphA2 (BD Biosciences, San Diego, USA). Biofunctionalization begins with immersing the metaEVchips in a 1 mM 11‐mercapto decanoic acid (MUA) ethanol solution (Cool Chemistry, Beijing, China) for at least 12 h. The metaEVchip was then thoroughly rinsed with deionized water (DW) to remove residual MUA and subsequently immersed in an activation solution containing 400 mM 1‐ethyl‐3‐(3‐dimethylaminopropyl) carbodiimide hydrochloride (EDC) and 100 mM N‐hydroxysuccinimide (NHS) (GreenSilver Reagent, Xiamen, China). After washing the metaEVchip surface with DW to remove excess EDC and NHS, the chip was incubated in 12.5 µg mL^−1^ anti‐GPC1 and anti‐EphA2 solutions in phosphate‐buffered saline (PBS) for 20 min. The metaEVchip surface was then blocked with a 50 µg mL^−1^ bovine serum albumin (BSA) solution in PBS (Sangon Biotech, Shanghai, China) to prevent non‐specific adsorption. Finally, the biofunctionalized metaEVchip was washed with DW to remove any unbound BSA, thereby completing the biofunctionalization of the metaEVchip.

### Clinical Testing

Serum samples were detected using the antibody‐functionalized metaEVchips to record the response of the nanoplasmonic biosensing spectra. These samples were obtained from the Department of Laboratory Medicine at the Fifth Medical Center of the General Hospital of the Chinese People's Liberation Army (Beijing, China) and the First Affiliated Hospital of Xiamen University (Xiamen, China). Serum specimens from healthy controls, patients with CCA, HCC, pancreatitis, breast cancer, colorectal cancer, and PDAC were collected with approval from the Ethics Committee.

### TEM Characterization of sEV from Serum

To characterize the morphology of purified sEV, carbon‐coated copper grids (400 mesh) were incubated with 10 µL of purified sEV sample for 1 min. The grids were then stained by incubation with 10 µL of uranyl acetate for 1 min. Excess stain was carefully removed, and the grids were air‐dried for 5 min at room temperature. The morphology of the purified sEV was subsequently observed using a transmission electron microscope (TEM) system (HT‐7700, Hitachi, Japan) operating at 100 kV, enabling high‐resolution imaging of sEV structures.

### Morphology Characterization of metaEVchip

Morphological characterization of the nanoplasmonic metaEVchip was performed using a TESCAN SOLARIS dual‐beam FIB‐SEM system (Czech Republic). The system enabled nanoscale structural analysis of antibody‐functionalized metaEVchips (anti‐GPC1/anti‐EphA2), specifically mapping sEV distribution patterns across nanostructured surfaces. Secondary electron imaging mode provided topographical data of sEV with 1.2 nm spatial resolution. MetaEVchips were prepared and processed for microimaging following the procedures outlined in a previous study25. Cross‐sectional views of the metaEVchip were acquired using FIB‐SEM at a tilt angle of 55° to provide a comprehensive view of the surface. Prior to FIB machining, a 1 µm thick protective platinum layer was deposited on the metaEVchip surface to prevent damage during milling. This protective layer helped maintain the structural integrity of the metaEVchip throughout the imaging and characterization process, enabling accurate and high‐resolution visualization of sEV and metaEVchips.

### Optical Simulation

Full‐wave simulations of the nanoplasmonic metaEVchip were performed using COMSOL Multiphysics 5.5 finite element software. In the simulations, 3D meta‐atoms with Floquet periodic boundary conditions were excited by normally incident light. An adaptive non‐uniform tetrahedral mesh with a minimum edge length of 0.5 nm was employed to ensure computational convergence. To model the detection of sEV, the biofunctionalized layer was approximated as a 20 nm equivalent dielectric layer, while the sEV were represented as dielectric nanospheres with diameters ranging from 40 to 160 nm. The refractive index of all bio‐nanospheres was set to 1.60.

### Data Collection

A total of 18 000 sets of spectral data were collected using the metaEVchip to detect GPC1 and EphA2 in serum samples, with 10 sets of spectral data obtained for each serum sample (five sets for GPC1 and five sets for EphA2). These data were divided into training and test datasets. Specifically, 10800 sets of samples were allocated for training (serum collected from the Fifth Medical Center of Chinese PLA General Hospital), 3600 sets were reserved for testing (also from the Fifth Medical Center of Chinese PLA General Hospital), and an additional 3600 sets were used as an independent external validation set (serum collected from the First Affiliated Hospital of Xiamen University and the South Hospital of the Fifth Medical Center of Chinese PLA General Hospital). The model was developed using the open‐source PyTorch machine learning framework and implemented in Python 3.8.

### Statistical Analysis

All spectral data were pre‐processed by baseline correction and normalized to the reference spectrum obtained from a gold mirror. Data were performed in quintuplicate, and results are reported as mean ± standard deviation (s.d.) to clearly indicate data variability. To assess the diagnostic accuracy of the PDAC assay, ROC curves and precision‐recall (PR) curves were constructed, and the AUC for both was calculated. Higher values of ROC‐AUC and PR‐AUC indicate better diagnostic performance of the corresponding models. All statistical analyses are performed using Origin software 2025 and Python 3.8.

### Ethics Approval Statement

The study was approved by the Ethics Committee of the Fifth Medical Centre of the General Hospital of the Chinese People's Liberation Army and the Ethics Committee of the First Affiliated Hospital of Xiamen University, with assigned project numbers KY‐2024‐4‐63‐1, KY‐2024‐10‐172‐1, and XMYY‐2024KY091, respectively. Written informed consent was obtained from each participant. Clinical serum samples were collected from the Fifth Medical Centre of the General Hospital of the Chinese People's Liberation Army and the First Affiliated Hospital of Xiamen. All aspects of the study met the standards set by the Declaration of Helsinki.

## Conflict of Interest

The authors declare that they have no known competing financial interests or personal relationships that could have appeared to influence the work reported in this paper. Correspondence and requests for materials should be addressed to Bo Li.

## Author Contributions

J.Z., Y.X., X.H., and Q.N. contributed equally to this work. B.L., J.Z., and BA.L. designed the study. J.Z. developed the concept. Y.X., S.L., and L.Z. collected clinical samples and data. J.Z. and Y.X. wrote the paper. J.Z., X.H., H.C., M.J., K.C., and J.S. obtained the nanoplasmonic biosensing dataset. X.H. and J.Z. wrote code and implemented the AI models. W.C., Y.Y., and Y.G. analyzed output data. J.Z., T.H., H.C., M.J., K.C., Y.D., and J.S. performed biological experiments and evaluations. Q.N. and Z.Z. performed a theoretical calculation. J.Z. conducted data visualization and made the figures. L.Z., Y.C., and B.L. gave clinical advice. S.L., Y.X., and L.Z. collected healthy control samples. B.L. and Z.J. conducted a funding acquisition. B.L. and Z.J. conducted project administration.

## Code Availability Statement

The codes that support the findings of this study are available from the corresponding author, B.L., upon reasonable request.

## Supporting information



Supporting Information

## Data Availability

The data that support the findings of this study are available from the corresponding author upon reasonable request.
