# Supplementary material for: Deep Learning‐Powered Nanoplasmonic Biosensing Approach Enables Ultrasensitive Extracellular Vesicles Profiling for Cancer Screening
Source: Adv Sci (Weinh). 2025 Sep 23;13(2):e11337. doi: 10.1002/advs.202511337 (PMC12786282; doi:10.1002/advs.202511337)
Supplement: Supplementary file 1 — Supporting Information [file ADVS-13-e11337-s001.pdf]

Supporting Information

Deep learning-based nanoplasmonic biosensing approach enables ultrasensitive extracellular vesicles profiling for cancer screening

Jiaheng Zhu†, Yingqi Xiao†, Xinyue Huang†, Qiang Niu†, Lihuang Zeng, Shaowei Lin, Mengqi Jiang, Tianhao Huang, Hanyang Chen, Yinong Xie, Yuan Gao, Wei Chen, Yiming Yan, Jiaqing Shen, Kaibin Chen, Yurong Dai, Zhipeng Zhang, Lijun Zeng, Yahong Chen, Boan Li, Jinfeng Zhu*, Bo Li**

This PDF file includes:

Figures S1 to S15
Tables S1 to S3

We characterized small extracellular vesicles (sEVs) purified by ultracentrifugation using transmission electron microscopy (TEM) and determined their various morphologies and sizes, as shown in Figure S1. These vesicles are typically round or rectangular in shape and usually have a size below 200 nm. This is consistent with the sEVs captured by our metaEVchip.

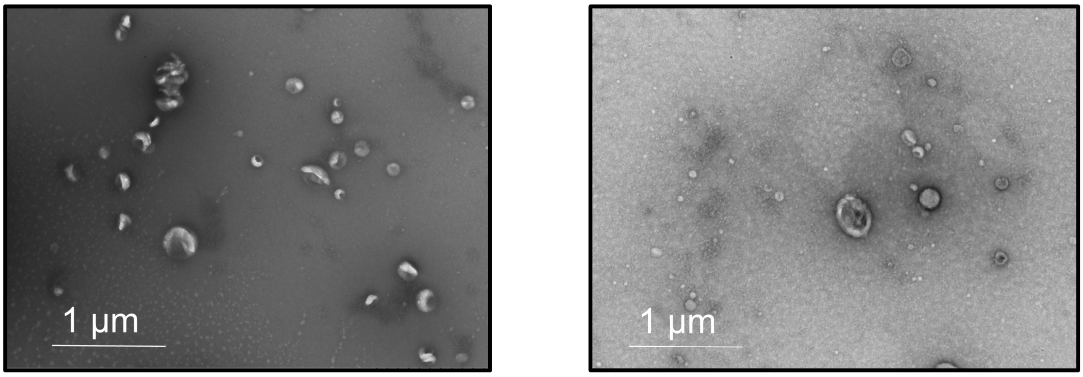

Figure S1. TEM characterisation of sEVs purified from PDAC serum samples by ultracentrifugation.

We evaluated the performance of the KAN neural network by combining it with four sensing modes in SE, RE, and ALE nanoplasmonic biosensing, respectively. The loss function was defined by calculating the difference between the model output and the actual label using binary cross-entropy loss (BCEloss). FigureS2 showed that the KAN neural network achieved the lowest BCEloss in both GPC1 and EphA2 detection.

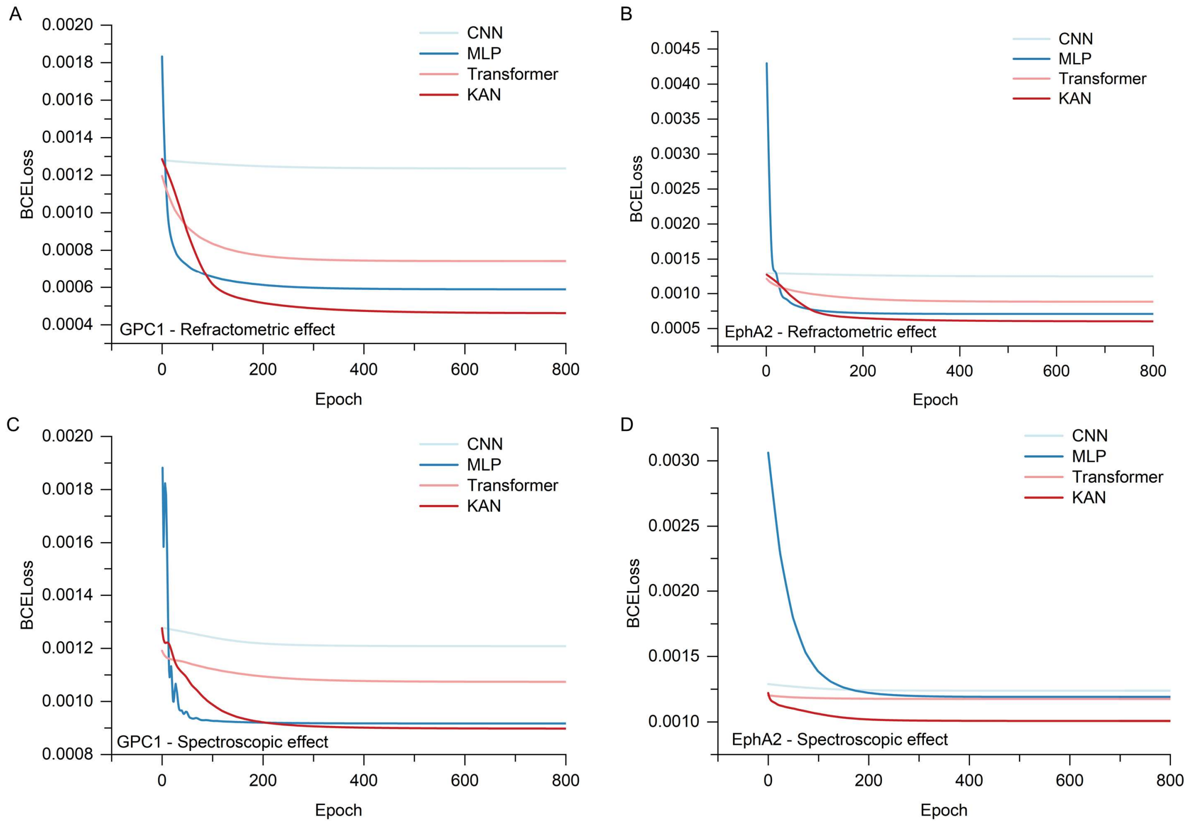

Figure S2. Learning curves of convolutional neural network (CNN), multilayer perceptron

(MLP), Transformer and KAN in RE biosensing with A) GPC1-metaEVchip and B) EphA2-metaEVchip. Learning curves of CNN, MLP, Transformer and KAN in SE biosensing with C) GPC1-metaEVchip and D) EphA2-metaEVchip.

To comprehensively evaluate the performance of the RE, SE, and NLE sensing processes integrated through the KAN neural network across four modalities, we compared the convergence behavior of the iterative loss function with other deep learning models. Analysis of the required epochs for loss function convergence revealed that the KAN neural network demonstrated the fastest convergence rate among all tested models, regardless of the sensing mode.

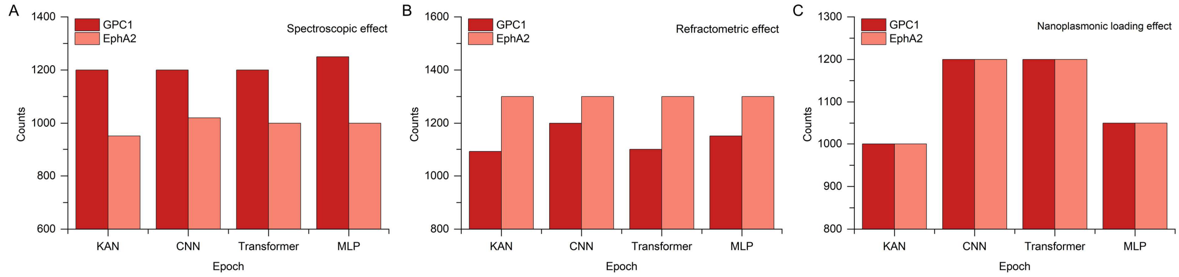

Figure S3. Number of epochs required for deep learning models to implement PDAC screening in A) SE, B) RE, and C) NLE sensing.

To better evaluate the performance of different deep learning models in achieving PDAC screening with traditional nanoplasmonic biosensing, we compared their training parameters, as shown in Figure S4. Among these models, the KAN model required the fewest training parameters. This efficiency can be attributed to KAN's unique architecture, which incorporates learnable activation functions based on spline functions. These functions allow KANs to achieve high accuracy with fewer parameters compared to traditional models like CNNs, MLPs, and Transformers. Additionally, KANs demonstrate faster convergence and improved interpretability, making them a promising alternative for complex tasks.

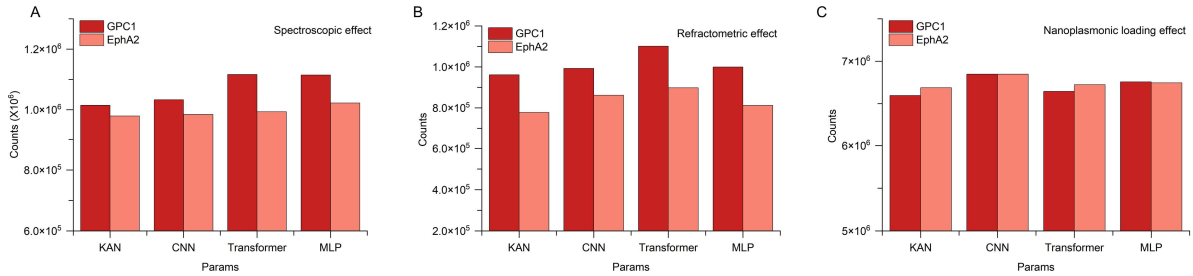

Figure S4. Training parameters required for deep learning models to implement PDAC screening in A) SE, B) RE, and C) NLE nanoplasmonic biosensing.

As shown in the Figure S5, as the number of iterations increases, the deep learning models continue to optimize, resulting in smaller loss values for both the training and validation sets. Once a large number of iterations are reached, the loss values in these sets stabilize across three traditional sensing approaches, indicating that the deep learning models have effectively learned and saturated most of the information from the training set. Meanwhile, the accuracy of both

the training and validation sets also stabilizes.

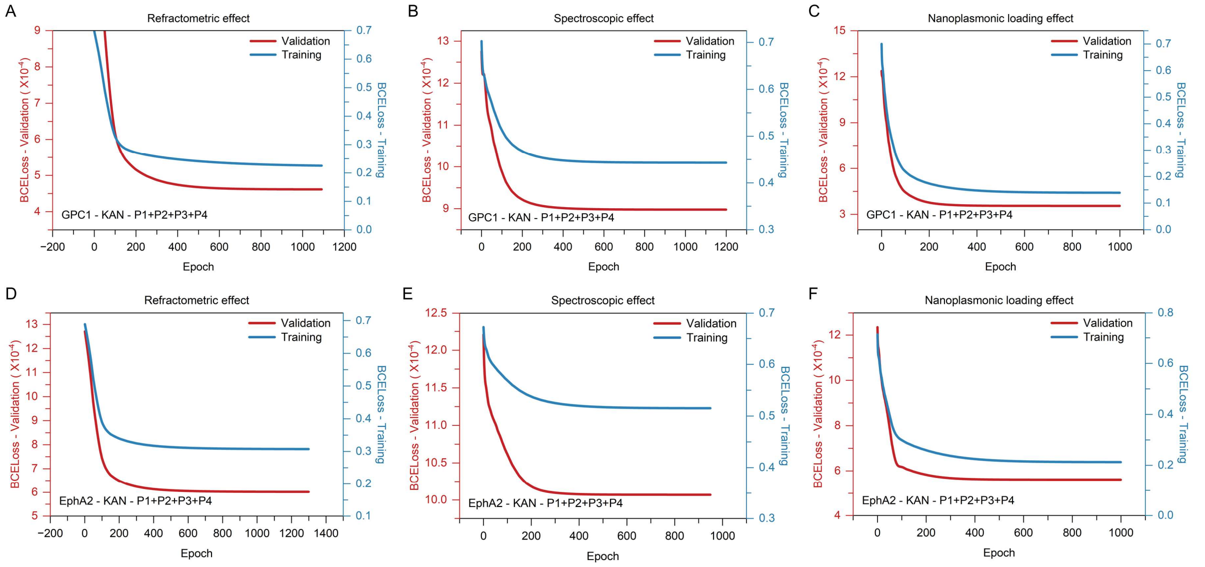

Figure S5. Loss function curves for the A) SE, B) RE, and C) NLE training processes during GPC1 detection are shown in Figure S5. Similarly, loss function curves for the D) SE, E) RE, and F) NLE training processes during EphA2 detection are also presented. These curves illustrate the behavior of the loss functions during training, which typically show a decline over epochs as the model converges.

As shown in Figure S6, the accuracy and AUC of SE, RE, and NLE quickly reach stable values as the number of iterations increases. This indicates that the KAN-based deep learning model rapidly mines a large amount of useful information from the traditional nanoplasmonic biosensing.

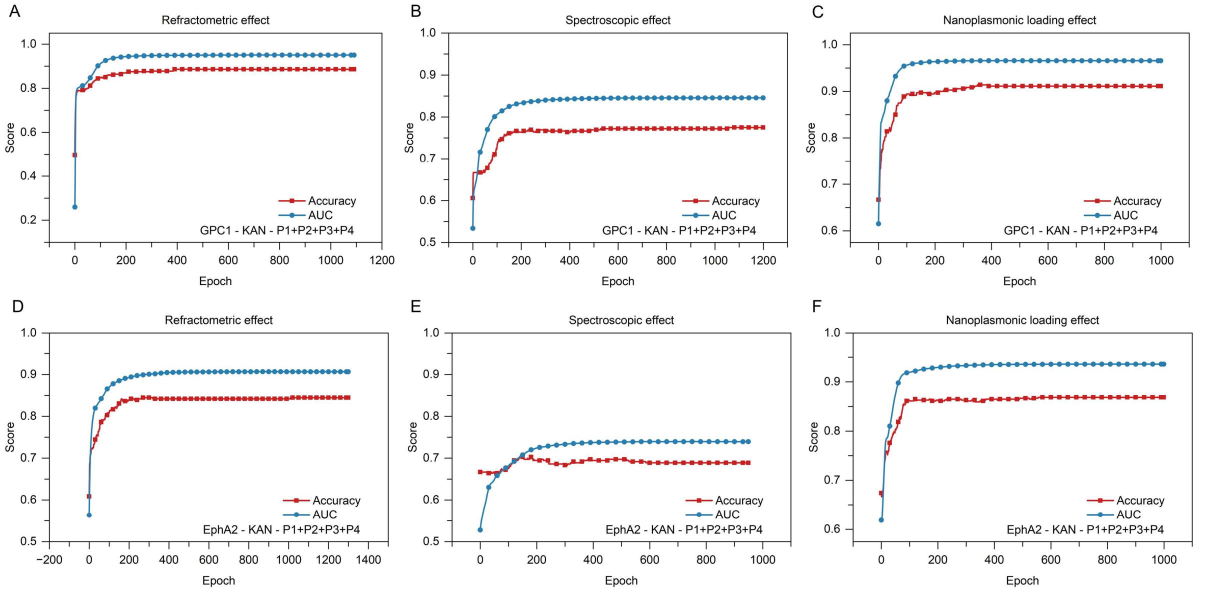

Figure S6. AUC and Accuracy score curves for the A) RE, B) SE, and C) NLE prediction processes for screening PDAC by detecting GPC1. AUC and Accuracy score curves for the D) RE, E) SE, and F) NLE prediction processes for screening PDAC by detecting EphA2.

To comprehensively evaluate the performance of the deep learning model combining four modalities for NLE sensing in PDAC screening, an accuracy-based confusion matrix was proposed. The confusion matrix provides a detailed overview of the classification performance, including true positives, false positives, true negatives, and false negatives, which helps in understanding the model's strengths and weaknesses. Notably, the KAN-based NLE sensing demonstrated the highest PDAC screening accuracy in both GPC1 and EphA2 detection.

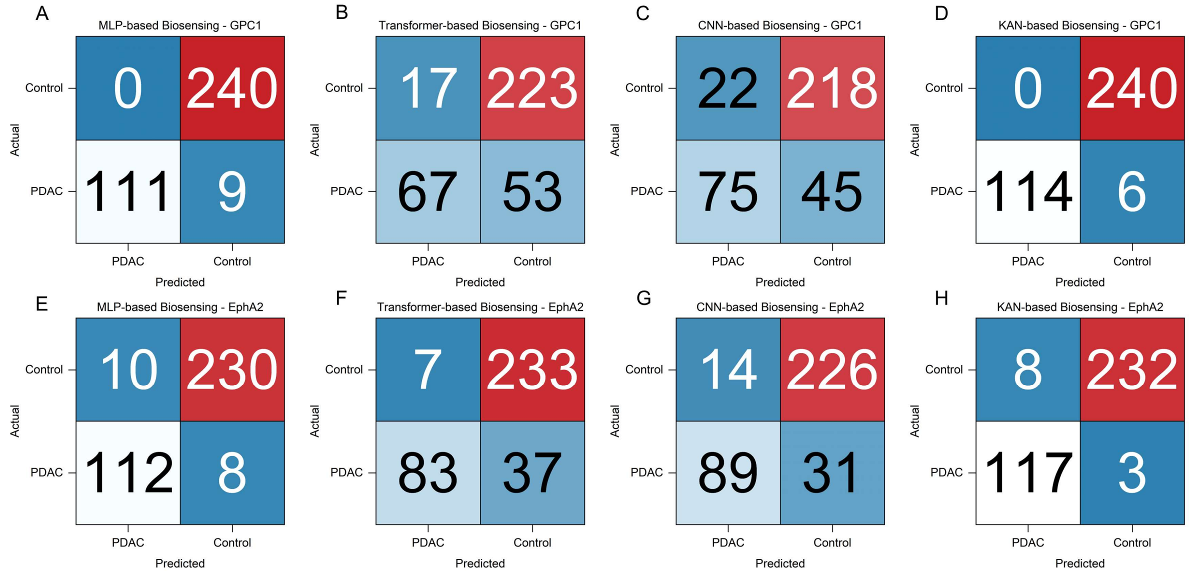

Figure S7. Confusion matrix for NLE sensing GPC1 by fusion of four modalities by A) MLP, B) Transformer, C) CNN, D) KAN. Confusion matrix for NLE sensing EphA2 by fusion of four modalities by E) MLP, F) Transformer, G) CNN, H) KAN.

Figure S8 shows that as the number of iterations increases, the accuracy and AUC of the deep learning model rapidly reach stable values. This indicates that the deep learning-based model efficiently extracts a large amount of useful information from the deep learning-based biosensing.

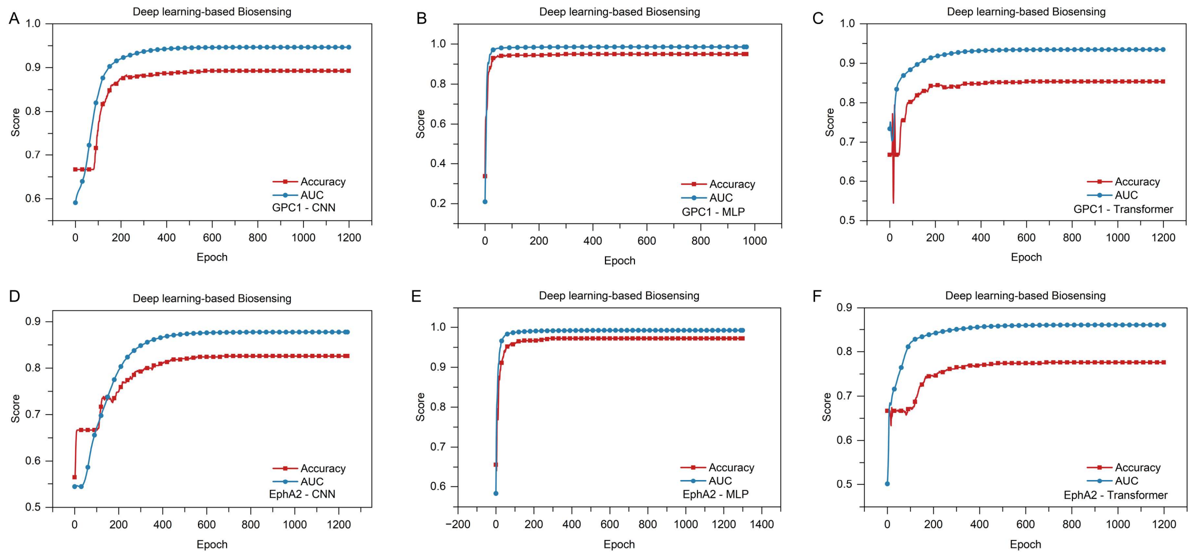

Figure S8. AUC and accuracy score curves for A) CNN, B) MLP, and C) Transformer prediction processes for screening PDAC by detecting GPC1. AUC and accuracy score curves

for D) CNN, E) MLP, and F) Transformer prediction processes for screening PDAC by detecting EphA2.

To better evaluate the performance of different deep learning models in nanoplasmonic biosensing, we compared their training parameters as shown in Figure S9. Among these models, the KAN model requires the least number of iterations.

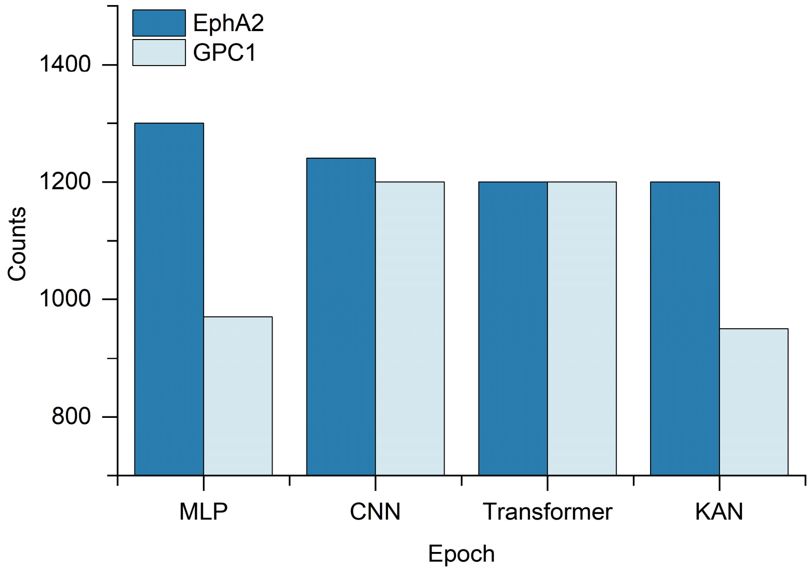

Figure S9. Epochs required by different deep learning models in nanoplasmonic biosensing.

To better explain why KAN-based biosensing has better diagnostic performance than traditional biosensing in the midst of PDAC screening, as shown in Figure S10, we introduce the following network architecture:

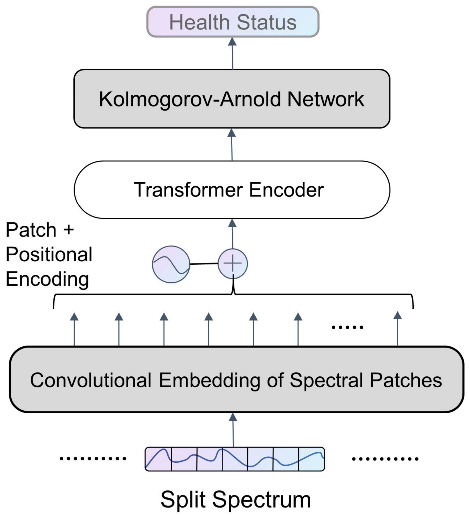

Figure S10. Explanation of KAN exhibiting high diagnostic in PDAC screening by position encoding neural network structure.

Figure S11 depicts the heatmap of attentional weights in the KAN-based nanoplasmonic biosensing implementation for PDAC screening. The visualization of attentional weights illustrates how the KAN model learns different spectral feature details, thereby enabling high

diagnostic performance in biosensing

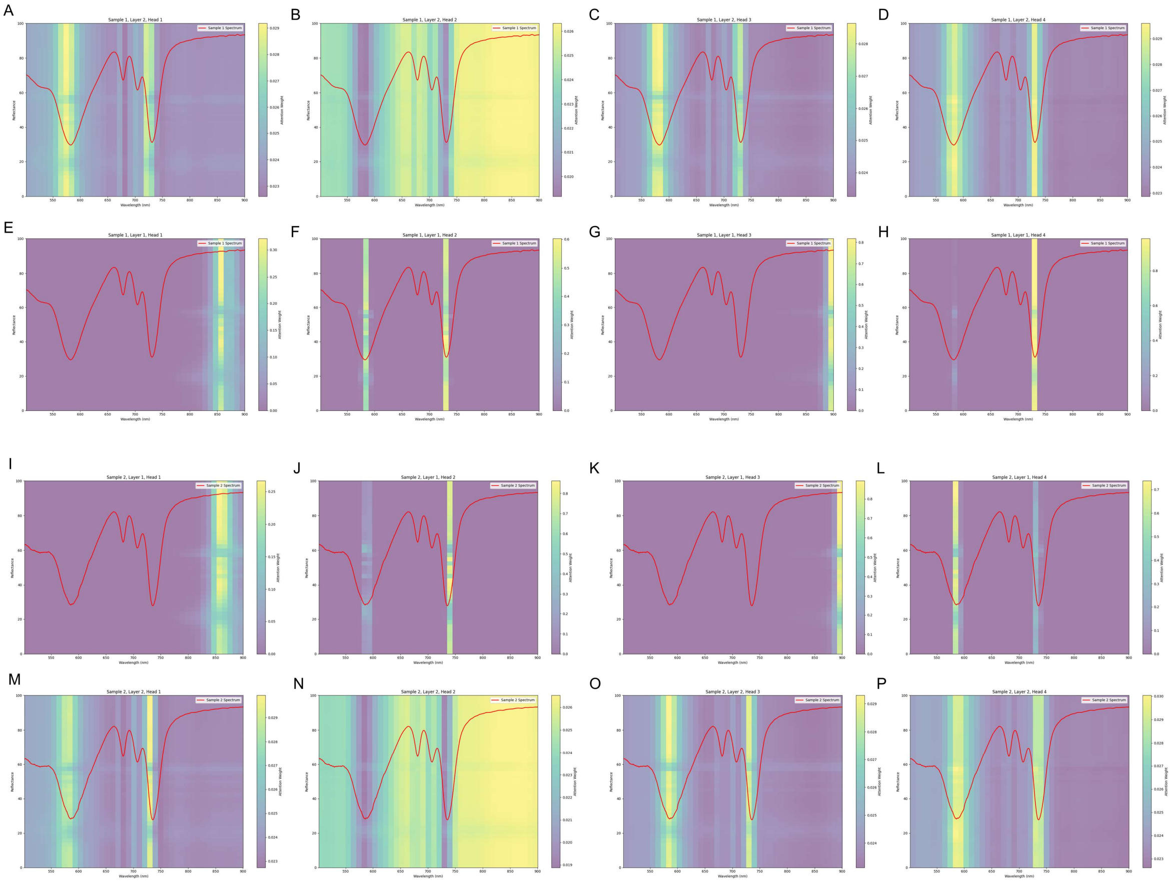

Figure S11. Heatmap of the attentional weights of different network layers and multiple heads of KAN in nanoplasmonic biosensing.

Figure S12 shows that the accuracy, AUC, PR-AUC and F1 of the KAN deep learning model quickly reach stable values as the number of iterations increases.

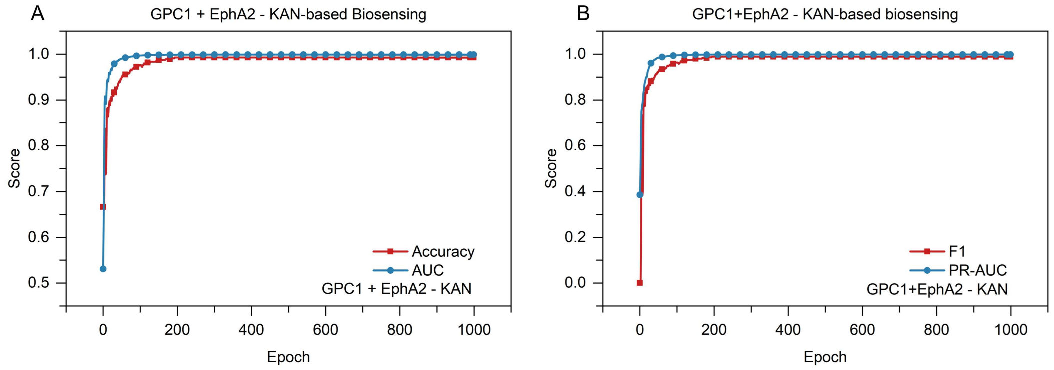

Figure S12. A) Scoring curves for AUC and accuracy of KAN-based nanoplasmonic biosensing for the combined detection of GPC1 and EphA2. B) Scoring curves for PR-AUC and F1 of KAN-based nanoplasmonic biosensing for the combined detection of GPC1 and EphA2.

The results demonstrate that the KAN-driven multimodal integration of GPC1 and EphA2 significantly outperformed single-analyte assays, achieving an AUC of 0.996 for distinguishing PDAC from benign pancreatic disease (BPD) (Figure S13A) and an AUC of 0.993 for differentiating PDAC from other cancers (Figure S13B) in an independent validation set.

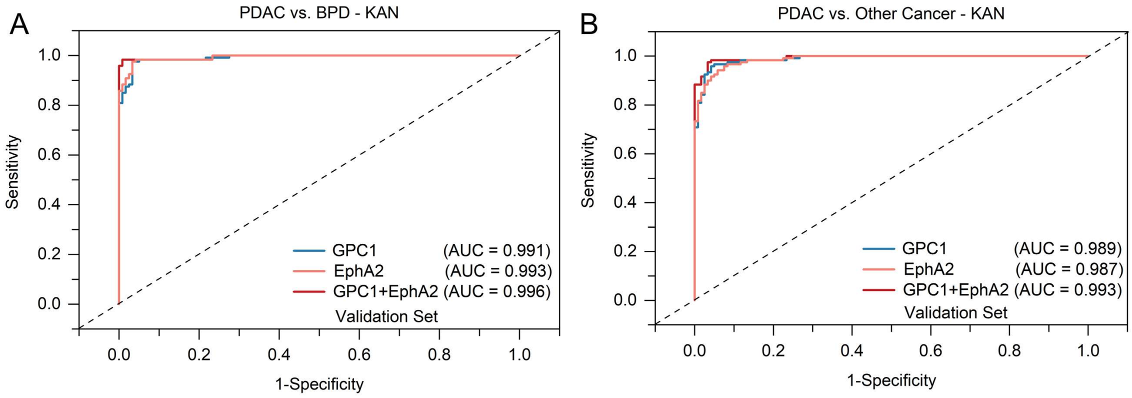

Figure S13. A) ROC analysis (KAN-based deep learning GPC1+EphA2): PDAC vs. BPD, B) ROC analysis (KAN-based deep learning GPC1+EphA2): PDAC vs. other cancers.

Figure S14 demonstrates that both clinical comparisons, all evaluated metrics—including specificity, sensitivity, and overall accuracy—exceeded 0.90.

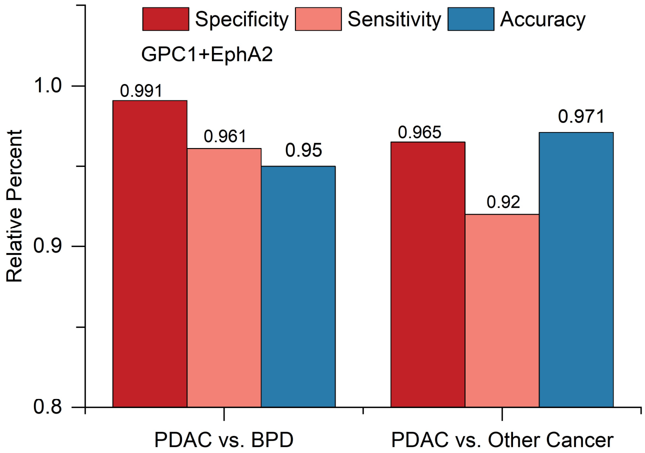

Figure S14. KAN-based deep learning delivers specificity > 0.90, sensitivity > 0.90, and overall accuracy > 0.90 across all evaluated subgroups.

The clinical readiness of our platform is evidenced by the wafer-scale manufacturability of the metaEVchip, which combines high production scalability (12-inch wafers) with exceptional uniformity (Figure S15).

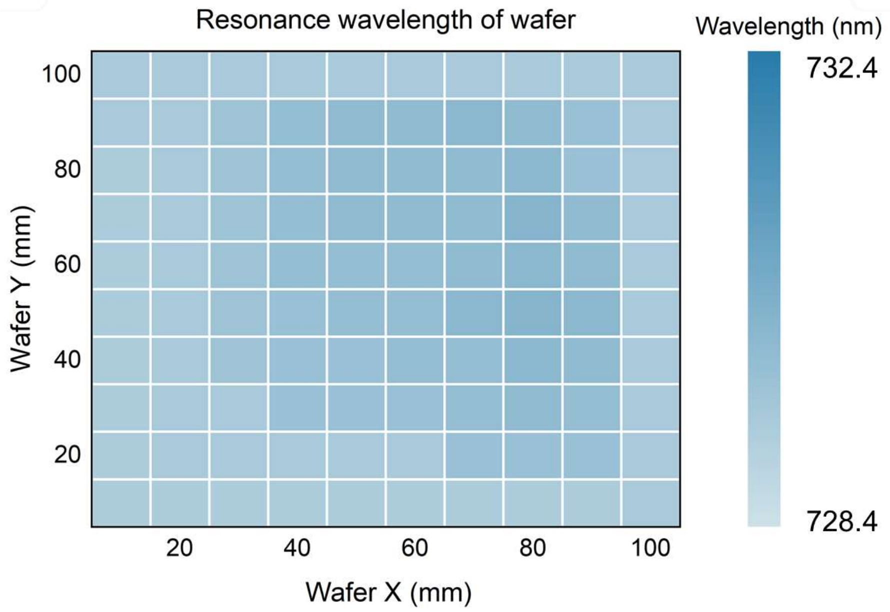

Figure S15. Uniformity of metaEVchip resonance wavelength.

Table S1. Performance comparison of different algorithms

	**KAN**	MLP	CNN	Transformer
Parameter Scale	**Small** **(Kolmogorov-Arnold theorem enables parameter-efficient compression)**	Small (Weak compression capability for high-dim data)	Moderate (Convolutional kernel stacking increases redundant parameters)	Large (Prohibitive compute overhead for multi-head attention and positional encoding matrices)
Interpretability	**High** **(Adaptive spline activation functions map physical significance. Attention heatmaps visualize multi-effect synergy)**	Low (Black-box model)	Medium (Black-box model. Convolutional kernels only capture local features)	Medium (Black-box model. Attention weights partially indicate spectral region importance)
Applicable Scenarios	**High-dim data** **Small/medium sample sizes**	Low-dim data Small/medium sample sizes	Image data	High-dim data Large sample sizes

Table S2. Comparison of detection between our work and other studies.

Ref.	Method	Chip Size	Sensing Direction	Clinical Validation Cohort Size
(21)	Plasmonic metasurface	3-mm-diameter	Dependent solely on λ-resonance shift (refractive sensing)	0
(23)	Plasmonic metasurface	2.5 cm × 2.5 cm	Dependent solely on λ-resonance shift (refractive sensing)	106
(24)	Plasmonic metasurface	3 mm × 3 mm	Dependent solely on λ-resonance shift (refractive sensing)	50
(40)	Plasmonic metasurface	3 mm × 3 mm	Dependent solely on λ-resonance shift (refractive sensing)	0
**This work**	**MetaEVchip + KAN-based Deep Learning**	**12-inch wafer**	**KAN-based full-spectrum analysis with multidimensional information for enhanced detection accuracy**	**1800**

Table S3. Performance comparison of KAN-powered deep learning biosensing under different biomarker combinations

	Comparative Groups	AUC	Specificity	Sensitivity
GPC1	PDAC vs. BPD	0.991	0.997	0.875
	PDAC vs. Other Cancers	0.989	0.997	0.871
	PDAC vs. EphA2-Positivity Cancers	0.988	0.982	0.895
EphA2	PDAC vs. BPD	0.993	0.998	0.887
	PDAC vs. Other Cancers	0.987	0.996	0.870
	PDAC vs. EphA2-Positivity Cancers	0.591	0.610	0.500
**GPC1+EphA2**	PDAC vs. BPD	**0.996**	**0.999**	**0.904**
	PDAC vs. Other Cancers	**0.993**	**0.998**	**0.883**
	PDAC vs. EphA2-Positivity Cancers	**0.991**	**0.982**	**0.889**
